# Participants in a peer-based nutrition and health program in Zimbabwe value dialogue, peer support, and tangible action: A qualitative exploration of peer group experiences

**DOI:** 10.1371/journal.pgph.0003525

**Published:** 2024-10-02

**Authors:** S. Riley Auer, Newton Matandirotya, Prince Mathe, Musawenkosi Moyo, Lisa Sherburne, Katherine L. Dickin

**Affiliations:** 1 JSI Research & Training Institute, Arlington, Virginia, United States of America; 2 Kgotso Development Trust, Beitbridge, Matabeleland South, Zimbabwe; 3 Department of Public & Ecosystem Health, Cornell University College of Veterinary Medicine, Ithaca, New York, United States of America; Maastricht University, NETHERLANDS, KINGDOM OF THE

## Abstract

Peer support groups are implemented globally, and viewed as cost-effective, scalable platforms for delivering health and nutrition programming. Quality is important for participation and achieving social and behavior change goals. Little research has explored the perspectives of peer group participants on quality. This manuscript describes community-based implementation research, and associated findings, which was conducted to learn how participants of a nutrition and health program define quality peer groups and how they suggest improving peer groups. In-depth interviews on experiences, benefits, and challenges were conducted with participants of health and nutrition peer groups, including group members (n = 64) and facilitators (n = 30), in three districts in Zimbabwe. Qualitative data were analyzed thematically and preliminary results were presented in six follow-up focus group discussions with interviewees to provide input on results and interpretation. Peer groups met some of participants’ needs for knowledge, social support, and visible improvements in their lives and homes. Participants described generally positive experiences that sustained participation and motivated behavior change. They highlighted group dynamics, interactive facilitation, and community recognition which support the credibility and motivation of group facilitators. Implementation could be improved by strengthening family engagement and more hands-on learning for encouraging participation. Local adaptation of group activities can address influences on behavior change and increase relevance to participants’ needs. The perspectives of core stakeholders are essential to understand what aspects of peer groups are most important to implement the approach with quality across contexts. Implementation research and continued monitoring to understand participant perspectives should be an integral part of all programs to ensure the application of adult learning principles and an appropriate balance between fidelity and adaptation for local relevance and engagement.

## Introduction

Despite some improvements in global rates of undernutrition in children under five, poor maternal and child nutrition continues to threaten health and development in low- and middle-income countries (LMIC), particularly in sub-Saharan Africa. Most trends are not on track to reach global nutrition targets [1]. In Zimbabwe, some progress has been made in achieving targets, such as exclusive breastfeeding of children under six months which has increased to 42%, but other indicators are not progressing. Still, 14% of children are born with low birthweight and only 11% of children receive a minimum acceptable diet [1, 2]. As in many LMICs, within-country data suggest nutrition inequalities; the highest rates of undernutrition in Zimbabwe are found in rural areas and in families in which women have little formal education [1–3].

The peer support group model [4–17] is a widely used community-based platform for reaching rural women to deliver locally relevant nutrition information and strengthen capacity to improve nutrition practices. Peer groups vary in size, composition, modalities, and focus areas. Within these groups, people of similar circumstances—in health and nutrition programming, usually women of reproductive age—come together to share their experiences, gain access to resources, and build knowledge, skills, and social networks [4, 9]. The underlying principle is peer-to-peer teaching to transfer health knowledge and attitudes, including social support [6].

A review of social and behavior change efforts of USAID-funded development food security activities identified that many programs use a participatory peer group model known as care groups; these groups “hold potential to be a shining star in a country’s health system, but only if done well [9].” Like other peer group models, the care group model relies on a cascade wherein a volunteer facilitator motivates mothers to adopt health and nutrition behaviors and, reciprocally, the group of mothers motivate the participation and leadership of the volunteer facilitator [10, 11]. Because the care group model has a specific structure and a clearly defined approach that is distinct from the program participating in this study, we use the term "peer group” throughout the paper unless specified by the literature or participant in direct quotes as a “care group.”

Evidence shows that peer groups can improve health and nutrition practices [4, 6, 8, 12]. An analysis of data from five countries [18] found greater reductions in under-five mortality and significantly higher coverage of child survival interventions in areas with care groups compared to areas without. Peer groups can be a cost-effective delivery strategy for expanding coverage of health and nutrition services using trusted peers in the same community, given that group leaders (facilitators) are unpaid volunteers and members of the community who are familiar with local families.

A major challenge lies in implementing interventions consistently with the quality needed to achieve and sustain impact [19, 20]. Implementation research exploring the experiences of participants—both group members and facilitators—can inform efforts to address implementation gaps that limit the impact of even interventions of proven efficacy. It is important to understand the participants’ perspectives and learn how to identify barriers, build upon strengths, and address challenges across contexts.

The literature on peer groups suggests several important areas of design and implementation to consider when examining the quality of group experiences: group membership and cohesion [4, 11], facilitation methods [6, 10, 19–21], attention to behavioral change [4, 5, 22, 24], and community linkages [4, 7, 22–26].

The make-up of peer group membership, and the ways that this impacts group cohesion and dynamics, are integral to the expected “support” function of groups [4, 24]. A model focused on true peers aims for minimal differences between members and/or members and leaders (facilitators), expected to ensure that group members feel a strong sense of belonging, are welcome, and feel safe to share. Group facilitation is also key [9] and peer-to-peer education is ideally engaging, dialogic, and experience-based, with problem-solving based on issues and solutions elicited from groups [10, 16]. Such experiential learning is considered essential for the development of critical and reflective thinking skills, unlike didactic learning which focuses on information acquisition alone [26]. To foster experience-based learning, it is important to train group facilitators not only on technical topics, but also on facilitation skills for interactive adult education and leading participatory activities [13, 27].

Attention to impactful behavior change pathways in curricula design and implementation also matters [4, 17, 25]. Most health and nutrition peer support groups aim to improve behaviors, and while peer interaction and problem solving is expected to motivate and support behavior change, the curricula must address relevant topics and actionable behaviors. Group sessions need to devote adequate time and attention to addressing factors that could influence behavior change, such as self-efficacy and access to health services, among others [8, 25]. Peer support groups can also provide opportunities to address the social determinants of health, the root causes of inequities that influence behaviors, including social norms and gendered power dynamics within communities and families in the local social context [28, 29].

Community linkages are fundamental for creating enabling environments [6, 25]. Broad recognition of the groups’ value promotes participation and sustainability, as well as supporting the motivation and credibility of peer facilitators. Peer group participants are embedded in family systems [30] and linkages with families, community leaders, and community members should be included at the design phase of the peer-based intervention [9].

A recent synthesis of literature found multi-directional drivers of motivations of peer group participants (i.e., volunteer group facilitators and group members), including resources provided by nongovernmental organizations, group dynamics, and mutual support as well as wider community support [22]. However, there is a dearth of research centering on how participants define a quality peer group experience, making it difficult to know what aspects of peer groups are most important and how best to implement this model across contexts. Reaching program goals of improved health and nutrition behaviors depends on understanding the experience of the participants expected to learn, adopt, and share locally, personally meaningful, and feasible practices.

Care groups were first implemented in Zimbabwe in 2014 and have been adopted by the national Ministry of Health and Child Care (MoHCC) [7]. Research from the Amalima program has shown promising practices and identified directions for improving peer groups [7, 14, 23]. This paper presents the results of implementation research on the follow-on program, Amalima Loko, a multi-sectoral USAID Bureau for Humanitarian Assistance (BHA)-funded Resilience Food Security Activity (RFSA) that also promotes health and nutrition through peer groups. The objective of this research was to understand member and facilitator perspectives on the quality of peer group experiences, the perceived benefits and challenges in the Amalima Loko community contexts, and how best to ensure high-quality experiences and the sustained participation needed to support nutrition and health behavior change. In this paper, “quality” refers to participant-defined quality of a care group experience. The acceptability, effectiveness, and sustainability of this widely used model depends on learning how to ensure implementation meets participant needs.

## Methods

### Intervention

In Zimbabwe, the peer support group approach has been adopted as an official strategy of the Zimbabwe MoHCC. The Amalima Loko program improves food security in five districts of Zimbabwe’s Matabeleland North region through increased food access and sustainable watershed management. The MoHCC and Amalima Loko refer to these peer support groups as care groups and they define specific roles for participants, which vary from those defined by other literature on the care group model [7, 10, 11, 14, 23]. MoHCC nurses work with program field officers to supervise village health workers who serve as Care Group Promoters (CGPs) and who, in turn, train and support the volunteer group facilitators, called “Lead Mothers.” Community leaders and CGPs select Lead Mothers and they receive training in group facilitation approaches and the health and nutrition messages endorsed by the national government. They invite pregnant women and caregivers of children under five years of age (called “Neighbor Mothers”) from 10–15 nearby households to join. Lead Mothers organize, mobilize, and facilitate regular peer group sessions with Neighbor Mothers to provide training and key messages promoting recommended maternal, child health, and nutrition behaviors; visit homes; and lead other activities in the community. Neighbor Mothers attend and participate in regular meetings, ask questions, and discuss topics. The groups benefit from layered multi-sectoral program delivery; groups are trained in agriculture and often linked to savings and loan and hygiene efforts, among others. This paper refers to Lead Mothers as facilitators and Neighbor Mothers are referred to as members; Lead Mothers and Neighbor Mothers are referred to collectively as participants.

### Study design

**Fig 1:**
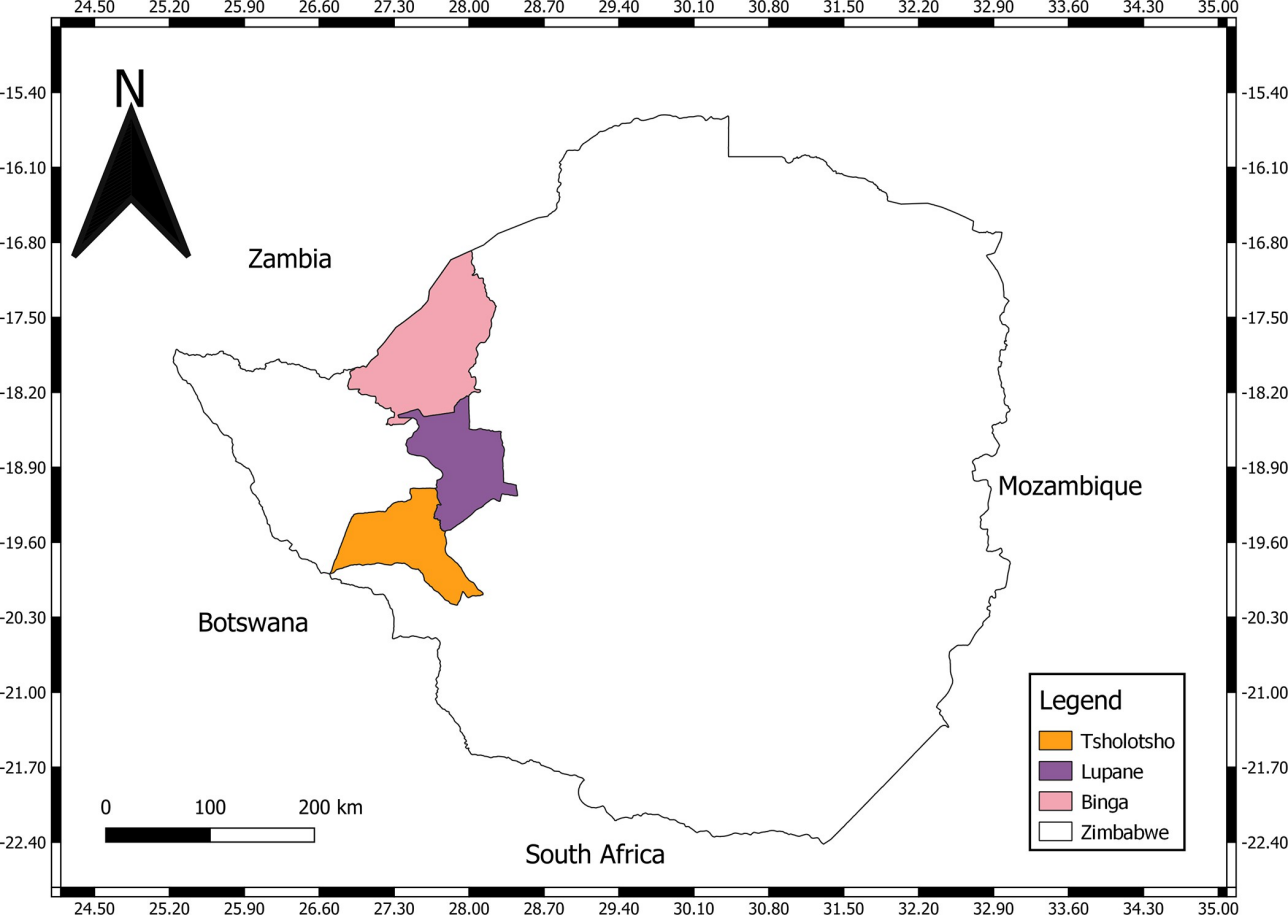
Study districts in Matabeleland North, Zimbabwe. Binga (pink), Lupane (purple), and Tsholotsho (orange) Districts in Matabeleland North, Zimbabwe.

This implementation research was conducted during September 2022 to January 2023 with women who were members and peer leaders (facilitators) of nutrition and health peer support groups implemented as part of the Amalima Loko program in Zimbabwe. Qualitative methods were used to explore the following questions in three districts of Matabeleland North (Binga, Lupane, and Tsholotsho) (Fig 1): [1] What characterizes quality peer group experiences as defined by group participants? [2] What challenges or experiences limit quality? [3] What do group members and leaders (facilitators) suggest to improve the quality of peer groups?

### Ethics statement

The research protocol received ethical approval from the Medical Research Council of Zimbabwe (MRCZ/A/2957) and John Snow, Inc. (JSI/22-70) in September 2022. Before each interview, enumerators explained the study purpose and the voluntary nature of participation, and sought and obtained written consent for participation and audio recording.

### Sampling and data collection

Three districts were selected to represent a range in cultural and contextual differences and stage of implementation: one district (Tsholotsho) had a long-running program, including implementation of the previous Amalima program in (2013–2020), and two districts (Lupane and Binga) had recently initiated groups through the Amalima Loko program. The low population density in Binga allowed an investigation of participation in a different program context characterized by widely dispersed dwellings.

Within three communities in each district, participants—both members and facilitators from multiple peer groups—were interviewed for a total of 64 members and 30 facilitators (Table 1). Subsequently, focus group discussions (FGDs) were conducted within the same sites and peer groups to gather input on the validity of findings and researchers’ interpretations across sites. In each district, one FGD was held with members (7 individuals) and one with group facilitators (5–6 individuals).

**Table 1:** Sample size for in-depth interviews and focus group discussions, by district.

In-depth Interviews	Binga	Lupane	Tsholotsho	Total
Group members (Neighbor Mothers)	26	19	19	64
Group facilitators (Lead Mothers)	13	10	7	30
***Interview total***	***39***	***29***	***26***	***94***
**Focus Group Discussions**				
Group members (Neighbor Mothers)	1 (7 people)	1 (7 people)	1 (7 people)	21
Group facilitators (Lead Mothers)	1 (6 people)	1 (5 people)	1 (5 people)	16
***FGD Participant Total***	***13***	***12***	***12***	***37***

*This sample comprised 131 participants across three districts.

Separate semi-structured, in-depth interview guides for participants were developed and translated. Interview guides explored participants’ perspectives on quality, benefits, and challenges, as well as their suggestions for improving the quality of their group experiences. Questions included, for example, “Can you describe what we would see if we visited a group session?”, and “what were memorable sessions and topics, and why?” Quality was explored with open-ended questions, and with more specific probes related to domains seen in previous research, including group membership and cohesion, facilitation, and community linkages. For example, “What characteristics of a care group make it high quality for you?” with probes “who is in the group?” and “who leads the group, and how?” among others. Interviewers explored lived experiences of peer groups, including care groups supported by the Amalima Loko program or others. The interview guides for facilitators also explored their roles, responsibilities, and experiences as Lead Mothers and reflections on group sessions and impact.

Interviews were conducted by trained field researchers proficient in the local languages (Tonga, isiNdebele) and lasted approximately 60 minutes. Participants were recruited and interviewed at program food distribution points that were convenient and regularly used for community meetings. Before each interview, enumerators explained the study purpose and the voluntary nature of participation, and sought and obtained written consent for participation and audio recording. The research team conducted the audio-recorded interviews, and transcribed and translated them into English. The research team provided participants with refreshments and U.S.$10 for transportation.

After coding and analyzing the interview data, six FGDs were held to share interview findings, check on variations across settings, gather input on interpretations, and refine recommendations to improve peer group quality. In each district, one FGD with five to seven individuals was held with group members and one with group facilitators; each included participants from multiple peer groups. Participants were compensated for their participation in the same way as interviews.

### Data coding and analysis

Data were coded by a team of four co-authors (SRA, NM, PM, LS), which included researchers involved in study design and data collection. Initially, SRA coded a duplicate transcript with each coder to ensure that the four interviews were coded by two researchers who compared and discussed coding to reach consensus and develop a shared codebook. Each coder then met with the codebook author (SRA) to discuss coding of initial interviews before being assigned several additional interviews. All transcripts were independently coded by two people and compared to ensure the consistent application of the code book, with meetings as needed to discuss questions or discrepancies and harmonize coding.

Interview data were coded according to dimensions of quality, challenges, and solutions using Atlas.ti, based on the study objectives. We began with codes derived from four dimensions identified in previous studies of (i.e., membership and group cohesion, facilitation, support of behavior change, and family and community support). Additional thematic codes arose during the coding process. These emergent themes can be characterized as: 1) locally-specific nuance to a quality dimension defined in existing literature, or 2) member-identified challenges (factors) and strategies for improvement (e.g., family support, appreciation for behavior change, etc.).

Themes were defined through a collaborative process between coders wherein the identifying coder proposed a thematic label and description, then discussed and revised the code until a consensus definition and label were defined.

## Results

Interview results are summarized in relation to the perceived benefits and challenges to participation and the four, predetermined quality dimensions that guided analysis—group membership and cohesion; group facilitation; support for the behavior change process; and community linkages—in addition to the emergent theme of family support. Where relevant, we note refinement of findings from FGDs. Selected illustrative quotes are included in the following sections, with the additional quotes in S1 Table.

### Benefits to participation

Participants described joining the peer group based on a personal relationship with the group facilitator, interest in gaining knowledge on specific topics (e.g., breastfeeding, child care, and hygiene), expectations for financial or in-kind benefits (i.e., access to income generation opportunities), and a desire for social support.

“I joined because I wanted to gain knowledge about caring for my child, what to feed the child, and how to keep the child healthy, as well as to learn about how to always be clean as women.” Neighbor Mother, Lupane

Most participants identified forms of social support as benefits, such as help from group members with household tasks and building hygiene structures, comfort in times of challenge, and reduced stress from talking together.

“We mobilize ourselves as a group to go around helping each other in painting and cleaning each other’s homes. We dug litter bins for each home of members in our group. We visit each other in their homes, teaching and reminding them what we have learned in the previous meeting.” Neighbor Mother, Binga

“The benefit, for now, is that I have people who visit me when [I] have challenges, who come to see me, and comfort me where possible.” Neighbor Mother, Lupane

Participants in Lupane and Tsholotsho also noted increased confidence and personal development as a key benefit of participation. Some expressed pride in being able to help others in their household and community with issues around child feeding.

Hygiene behaviors and vegetable gardening interventions are appreciated by group members, but are delivered by other platforms within the program. Nearly all participants mentioned access to income generating activities because the program offers groups opportunities to engage in other program activities, such as agriculture training and village savings and loan (VS&L).

### Challenges

Group facilitators and members in every district frequently mentioned low attendance and lateness to group sessions as challenges. These were often attributed to a lack of family support for women to take time away from household tasks.

“I’ll usually ask my husband to go and attend these meetings so if he allows me, and then people delay coming, it becomes a challenge because I will then go back home late and this causes arguments between me and my husband.” Lead Mother, Lupane

Additionally, some responses suggested that groups wait to begin a session until all or most of the expected members arrived, delaying everyone in returning home. In Binga, where homes are widely dispersed, travel to group sessions made attendance challenging. Although some participants in all districts shared concerns about distance, FGD participants felt that it is a barrier primarily in Binga.

Some participants mentioned times of internal conflict in their group, but noted that the facilitators and CGPs have skills to resolve conflicts. Some shared that grandmothers, as respected elders, can help also.

“I have had conflicts within the group where some women were fighting but the Promoter [CGP] managed to sit them down and emphasize working together because if there are conflicts there is no going forward, but forgiving each other is important.” Lead Mother, Tsholotsho

*“*Grandmothers assist us when there is a conflict in talking. They can say, ‘that is not the way to talk just because the one who is correcting you is an older figure,’ then we will listen especially to the young mothers.” Neighbor Mother, Lupane

### Group membership and cohesion

When asked about similarities and differences among members, the member characteristics mentioned most often were source of livelihood, religious affiliation, marital status, and age. Participants reported that their group members were all female, and most were pregnant or lactating mothers meeting program eligibility requirements. Some groups also included several grandmothers as primary caregivers of young children. The ages of group members varied; some groups included people ranging from 17–67 years, while others had members aged 25–35 years. Most expressed appreciation and/or perceived value in mixed-age groups.

Socio-economic characteristics also varied somewhat between and within groups. In some groups, members were all landholding and of the same faith, while other groups were more diverse. Participants expressed positive views on both more uniform and diverse group compositions. Rather than focusing on similarity to peers, quality group membership was often described in terms of responsiveness to members’ needs and positive, inclusive interpersonal dynamics.

“A quality peer group is one that takes into consideration the needs of its group members. A group that is inclusive in nature—including everyone in the community despite their social status. A high-quality group should be characterized by high levels of respect for each member considering that we are all adults in this group, so we need to be respected.” Neighbor Mother, Binga

Participants talked about a sense of shared identity and belonging with an emphasis on unity, harmony, respect, and love within groups, as well as a willingness “to learn and advise each other” yet be “humble enough to find out information they are not sure about.”

Most participants were satisfied with group dynamics, despite some internal conflicts as noted under the section on challenges. Groups in Tsholotsho reported positive interactions between group members and shared many examples to demonstrate the strength and longevity of these social bonds. In Binga and Lupane, where groups were newly formed, participants made fewer references to members of the groups being friends.

### Group facilitation

Members frequently mentioned enjoying active learning methods, such as singing and dramas—activities included in the Amalima Loko program to increase participatory engagement. There was interest in expanding the amount and variety of participatory activities, such as storytelling and creating dramas, as well as practical learning activities. Responses also indicated that the primary instruction format often involved facilitators reading directly from their manual or notes. A few facilitators wished that members would bring topics or points for discussion, suggesting that these facilitators found it challenging to start a dialogue.

When asked about memorable learning, members recalled specific occasions when they learned skills by doing and when they felt acknowledged by the facilitator for practicing the skill correctly or providing relevant information or experiences.

“I felt supported by the facilitator and other members when we discussed the weighing and oedema measurements and later practiced, and the facilitator acknowledged that we did it the right way.” Neighbor Mother, Tsholotsho

### Appreciation for behavior change

Most members appreciated the changes they see as a result of participation in the groups and felt that this represented a quality group experience. Participants highlighted concrete evidence of change, such as “toilets, pot racks, improved health and child care among other things” and “smartness,” meaning homes that look clean and hygienic to visitors and community members. Group activities that led to visible changes in households and communities, particularly related to hygiene and breastfeeding, were most often mentioned as memorable and favorite topics.

“The way of drinking water has changed as each person now has his or her own cup for drinking water. We also wash [our] hands after using the toilet using the tippy tap. The toilet itself is a result of the group and so is the rubbish pit, pot racks, and clean yards.” Neighbor Mother, Tsholotsho

Some members felt proud to be able to share new knowledge and skills with their family and friends.

“I taught my friend how to breastfeed her child whilst she is seated because she was doing it wrong compared to what we have been taught in the care group.” Neighbor Mother, Binga

Group facilitators also said that, ideally, a quality group was one in which members would “practice what they have been taught.”

The most common suggestions strengthen sessions to achieve greater behavior change related to tailoring the content so that it is relevant to the age and stage of development of group members’ children. Members suggested additional topics of interest, some of which were beyond the scope of health and nutrition: VS&L activities (such as soap and lotion making), family planning, HIV/AIDs, sick child care, and topics for children over five years of age.

### Community linkages

Many participants noted that the groups are recognized by communities because community leaders select group facilitators and link groups to community structures through the CGPs. Additionally, many said that community members appreciate groups for contributing to community development through hygiene and various other ways. Some reported that community members and leaders observe or contribute to peer group meetings.

“Yes, [the community] realizes what we are doing and the village head provides us with information about what is going to happen in the area, and other members comment on how the existence of the group has elevated the community… As Lead Mothers, we were chosen in a community meeting, so the community is aware of the existence of care groups.” Lead Mother, Tsholotsho

Most facilitators, particularly in areas such as Tsholotsho where peer groups were previously established, felt proud of their role. They mentioned signs of increased social status, such as recognition by community leaders, invitations to speak in community meetings, and appreciation from the community. However, some mentioned hearing gossip mocking facilitators for doing the work of village health workers (i.e., growth monitoring) without compensation.

### Family support

Lack of family support emerged as a primary barrier to attendance. In areas where this or another program previously implemented peer group activities, many participants reported family approval and even enthusiasm for their participation which was attributed to the visible improvements in households and children’s hygiene. In new program areas, however, family approval for attendance was a common challenge; some questioned the benefits of participation and others preferred the woman focus her time on household tasks. Some facilitators reported visiting families to encourage participation and to reduce absenteeism and tardiness, but this increased their workload and is not a planned program activity. Some members invite their husbands to join to enable attendance.

“The most common challenge among care groups is men who do not allow their wives to attend meetings. But with our care group we do not face such a challenge because we visit the group member the day before the meeting and inform them in the presence of the husband that we will have a meeting and inform them about the lessons that we are going to be having so that the husband gets to know about the program as well.” Lead Mother, Lupane

Some participants also noted that family support, especially from mothers-in-law, is critical to trying recommended nutrition behaviors they discuss in groups, especially related to infant and young child feeding.

*“*In most households, the young mothers especially have their mother-in-law who wants to feed the grandchildren some food items instead of them practicing exclusive breastfeeding.” Lead Mother, Tsholotsho

## Discussion

Peer support groups are a core program strategy for social and behavior change interventions designed to improve health and nutrition outcomes among pregnant and lactating women and caregivers of young children [9–12, 22]. This implementation research explored group member and facilitator perspectives on dimensions of quality in a peer group intervention within the Amalima Loko program in Zimbabwe. From the perspective of participants, a quality peer group is one that reflects strong social bonds between members, engages members in activities and dialogue, and leads to visible improvements at home and in communities. Most participants reported positive experiences, and benefits were more salient in districts with long-standing versus newly formed groups. Challenges were largely related to absenteeism or tardiness. For members and facilitators alike, community and family decision makers influence whether they join groups and attend sessions, and whether they can adopt improved health and nutrition behaviors. Suggestions to improve quality included more family engagement, hands-on activities, and interactive activities.

Experiences reported by group members and facilitators indicate that implementation varied and, sometimes, deviated from program guidelines in response to aspects of context, membership, and leadership, illustrating the challenges of balancing fidelity to program design with adaptation to local conditions [19, 31, 32]. Fidelity is the degree to which a program is implemented as intended by program developers [19]. Adaptation often refers to changes that are made when a program is implemented in a new setting to adjust to country or regional differences. However, adaptation can also happen at the level of program implementers who interpret program guidance in accordance with their own views of what is effective, feasible or convenient. In this way, front-line facilitators may act as “street-level bureaucrats” [33] who shape programs in ways that reflect their values, training, accountability, and the challenges they encounter. Thus, decisions by individual implementers can greatly impact participants’ experiences in decentralized models, such as peer support groups.

Adherence to key aspects of protocol is essential to convey the correct information and ensure accountability to program goals and procedures, but appropriate adaptation to respond to the needs and conditions of participants can potentially support effective and sustainable implementation [31]. Peer support groups offer an important context for considering this balance: understandably, the training of facilitators often focuses on providing knowledge and encouraging fidelity to program messages and materials. Yet, by its very nature, implementation through a local peer with lived experiences similar to what participants offer, is likely to result in some level of appropriate adaptation that can contribute to perceptions of quality.

As expected, group cohesion was key to benefits and those members who had participated for long periods reported a shared sense of belonging. Social bonds and friendships within peer groups can significantly impact the adoption of recommended behaviors and provide benefits beyond nutrition and health [24]. Participants often appreciated variation in member characteristics because people with different experiences can support each other across life stages. This can be seen as an adaptation of “peer” to include members such as grandmothers who vary in age from the primary target group. On the other hand, including “non-peers” of differential status could be problematic if they dominate the group or share inaccurate information [26]. Group facilitators have autonomy to invite members such that their close associates could be more likely to be included and some community members could be left out.

Participant preferences for participatory approaches, such as songs and drama, and their recollection of learning related to sharing experiences and working together align with principles of adult learning, such as collaboration, building on lived experience, mutual respect, self-direction, and learning by doing [26]. While group facilitators receive training in dialogic methods, these may be new and challenging approaches for some [9], suggesting an opportunity to increase dialogue by adjusting the sessions to have more time and space for problem solving. Capacity could also be strengthened by modeling the participatory approaches during training sessions and by additional support for developing relevant skills, such as peer mentoring.

Applying the principle of self-direction in adult learning could ensure that the curriculum content and focus for behavior change aligns with members interests and is relevant to the ages of the children they care for and other household characteristics. Given the timing in new program areas in this study, it was difficult to assess participant views on the range of topics included under Amalima Loko. Members noted that they received a lot of information on breastfeeding, but this was not relevant for everyone. Many expressed an interest in hygiene to generate tangible improvements in home environments. Allowing members to choose areas of learning and address specific needs to improve behaviors within a set of relevant topics and engaging materials may be an adaptive strategy that achieves greater participation and impact than asking all groups to follow a structured and detailed curriculum. Program designers could identify the core elements for fidelity and acceptable variations, and train and support facilitators in thoughtful adaptation [19].

Group facilitators appreciated recognition in the community which motivated and empowered them, and facilitated their role in creating effective groups. Connections to community leaders enabled information exchange so that peer groups could learn about what is happening in the community, and the broader community could learn what peer groups are doing and access health and nutrition information.

Participants raised challenges related to their context and daily lives; these challenges raised the role of structural and social determinants of health. Specifically, the importance of family engagement emerged through the research, which is consistent with global recognition that peer group participants are embedded in family systems [30, 34]. Given family roles and gender norms in the context of rural Zimbabwe and many other settings, family support is essential to facilitate women’s ability to participate, as well as to put recommended nutrition and health behaviors into action [34, 35]. Some individuals participating for long periods noted that actions taken at home by participants provide an opportunity to increase the perception of program benefits, enhancing some families’ willingness to allow women to attend. Community-level strategies to highlight these visible benefits of peer group participation present an opportunity to create more support for peer group participation, as well as social and behavior change.

While impacts on behavior change were not the focus of this implementation research, these findings demonstrate that participants do feel peer groups have led to some behavior change. Group facilitators described making and sustaining the most behavior change. This reflects the benefits of teaching as a mechanism for adult learning and behavior change, and implies that an evolving structure of the peer group model (i.e., members becoming group facilitators and starting new groups) would facilitate learning and strengthen self-efficacy.

Amalima Loko is a multifaceted program. In their responses to questions about nutrition and health peer groups, participants did not always distinguish these activities from other program components such as community health clubs and VS&L groups. A lack of demarcation between various Amalima Loko activities may have resulted in the conflation of views on these other activities with those of the health and nutrition-focused peer groups. While challenging for researchers, this lack of demarcation is not negative from a program implementation perspective. For example, findings suggest that peer group participants recognize that their peer group membership opened additional opportunities and may increase interest in participation among eligible members, as well as family approval of participation. Quality peer group experiences feed into the larger program because groups are used to deliver multiple activities.

The qualitative interviews and FGDs described in this paper constituted an exploratory phase, which fed into a human-centered design (HCD) approach to co-design solutions to identified challenges. The findings discussed here convey peer group participants’ priorities, challenges, and suggestions. These were synthesized into HCD tools and shared back to program staff anonymously in workshops designed to co-create solutions and discuss their feasibility and relevance. Co-created solutions were then tested and refined in collaboration with communities in subsequent phases of the HCD process.

### Strengths and limitations of the research

A key strength of this research is the focus on understanding program quality from the perspective of key stakeholders during the implementation period. Gathering the perspectives of group members and facilitators, and including both long running and recently initiated groups, was informative and provided diverse viewpoints. These stakeholders are often overlooked in design phases, and it is essential for effective, sustainable programs and engagement of communities to gain these perspectives during implementation on what is or is not working.

The results presented here provide insights that can be helpful for social and behavior change nutrition program design implemented through peer support groups.

This implementation research was designed to gather input from participants on their perceptions of the quality of on-going program activities, and as such, the design revolved around capturing what was currently happening. For this reason, data collection focused on participants rather than a representative sample of women in these communities. We could not reach women who had not participated in peer groups or who had dropped out. Interviews with women who did not participate, or women who had dropped out, might have been valuable for understanding additional challenges such as any negative group interactions and relevance of topics, among others. However, such women may have been reluctant to speak forthrightly due to social desirability since it could impact relationships with neighbors. Additionally, the scope of this research focused on the perspectives of those participating in care groups.

The focus on current implementation of a multi-year program also meant that there were different stages of implementation across districts as the program rolled out over time. The intention was intended to be as flexible and participatory as possible, to allow for adaptive learning on what peer group members valued and perceived to be important for a quality experience as well as their suggestions and priorities for improving implementation and quality. Follow-up FGDs were useful for checking whether findings from one district applied to other communities and for gaining additional insights to guide and refine interpretation.

It was notable that when asked about how to improve the quality of peer groups, the group participants in FGDs tended to focus more on how *they* could do better, rather than how the program or facilitator could do better, referring to the need to be more accepting of group members or take more steps to improve their behavior. This may reflect cultural and gender norms in this setting or other personal values. However, as in all research asking people to critique a program in which they participate, it is possible that, to some extent, responses reflect social desirability and concern for the feelings of those implementing the program.

In summary, members appreciate peer groups for gaining practical information, skills, and pride, and group membership offers benefits for women beyond nutrition and health. It is important to consider how to sustain this cohesion, even after women have completed the curriculum. Examples in Amalima Loko include groups focused on VS&L or other beneficial activities, but input from stakeholders could identify other priorities. Balancing fidelity to peer group program goals, methods, and curricula with opportunities (and capacity strengthening) for facilitators to adapt to the context and interests of group participants is important for effective implementation of peer group models [31, 36]. If well managed, such adaptation could support the application of adult learning principles by giving members choices about topics and activities, having them lead discussions or develop dramas, facilitating discussion among the different group members with similar situations, such as age of child, and allowing time for reflection and problem solving related to behavior change.

## Conclusions

Program design and implementation for widely used peer group models should be based on understanding the quality of experiences from the perspective of participants and other key stakeholders. In Amalima Loko, active participants reported appreciating new knowledge and skills and often emphasized the value of support from their group, preferring dialogic and participatory activities. Participants want to improve their homes and communities, so tangible change in behaviors, such as those related to hygiene like tippy taps and latrines, were valued. Visible changes contributed to recognition of groups as community resources and are likely to benefit the sustainability of groups. While peer support groups are implemented widely as a cost effective and scalable approach to meet behavioral objectives, research on quality experiences from the perspective of members themselves is useful to learn what aspects of peer groups are most important to implement this model across contexts. Designers and implementers can identify essential elements for quality implementation combined with adaptation to context *based on inputs from participants* to achieve the full potential of the approach. Implementation research and continued monitoring to understand participant perspectives on quality should be an integral part of all large-scale peer group programs. This will ensure the application of adult learning principles and an appropriate balance between fidelity and adaptation that meets the goals of the program and the participants and other key stakeholders.

## Supporting information

S1 Checklist Inclusivity in global research. (DOCX)

S1 Table Additional illustrative quotes by theme. (DOCX)
