# Supplementary material for: Participants in a peer-based nutrition and health program in Zimbabwe value dialogue, peer support, and tangible action: A qualitative exploration of peer group experiences
Source: PLOS Glob Public Health. 2024 Oct 2;4(10):e0003525. doi: 10.1371/journal.pgph.0003525 (PMC11446453; doi:10.1371/journal.pgph.0003525)
Supplement: S1 Table — (DOCX) [file pgph.0003525.s002.docx]

**Table 2: Additional illustrative quotes by theme**

| **Theme** | **Quotes from Interviews** |  |
| --- | --- | --- |
| Benefits | “We support each other, we can give them money if they need it, if someone is sick, we help each other by fetching water and cooking for them…” Neighbor Mother, Lupane  “Care groups enable us to comfort each other, relieve stress and forget about our own problems. The good thing about care groups is that we have become more developed and now we have become cleaner and more informed.” Neighbor Mother, Tsholotsho  "We have succeeded in sharing knowledge around issues that affect us as caregivers of under fives. We now have access to labor in various labor demanding activities. We have also capitalized on working together and became successful in building various structures, including cupboards.” Neighbor Mother, Lupane  “I have benefitted through empowerment. I got information about health and hygiene, which is helping to take care of my baby and home. The selling of maize has increased my income and I have bought some of the utensils I did not have at my home.” Neighbor Mother, Binga | |
| Membership/Cohesion | “Young mothers talk about cleanliness and drama in the drama, whilst grandmothers sing, and teach us about how they did things way back compared to how things are done now.” Neighbor Mother, Lupane  “Yaaaaa, they have been so supportive to me personally considering that I was struggling on digging the pits for throwing litter but with the help of these people I have managed to get help.” Neighbor Mother, Binga  “Since the elders have more knowledge, the grandmother teaches us about caring for the kids, as well as ourselves as mothers, and hygiene is emphasized by the grandmother. Young mothers participate by acting and singing in relation to the topics being learnt.” Neighbor Mother, Lupane  “Love and unity should prevail in the group so that people can easily participate without any fear and feel that they belong to the group.” Lead Mother, Binga |  |
| Group Facilitation | “Interactive activities, such as drama and song, help us understand better and we also share with other family members as well as our friends.” Neighbor Mother, Lupane  “We are allowed to participate and to comment, airing our views and participating without any challenges. We all come up with solutions if a problem is identified and we agree on what is done.” Neighbor Mother, Binga  “I and other members of the care group spoke and shared a lot when we discussed pot rack designs with other care groups.” Neighbor Mother, Tsholotsho  [The lessons are practical to apply at home], because we are taught and we demonstrate how to do it and how it can be done.” Neighbor Mother, Binga |  |
| Appreciation for Behavior Change | "We now clean the kitchens unlike before and we have built wooden racks (ingalane) to dry our plates.” Neighbor Mother, Lupane  “The group has benefited me in a number of ways as I have managed to get information about hygiene and health. Through the teachings, I have now pitched my tippy tap (SiGubhu Giya) for washing hands after using the toilets. Also, I now have pits for litter.” Neighbor Mother, Tsholotsho  “I used to stay in a dirty place, but it has changed, I am [now] comfortable to be in a clean environment. We are also taught how to relate with those we stay with. I used to wait until my baby cries before I breastfeed but now I know I have to breastfeed frequently. Neighbor Mother, Lupane  “I did not have a toilet before I joined the care group but now I can proudly say that I own a toilet.” Neighbor Mother, Tsholotsho  “I taught my friend how to breastfeed her child whilst she is seated because she was doing it wrong compared to what we have been taught in the care group.” Neighbor Mother, Lupane |  |
| Community Linkages | “[Community leaders] recognize what we are doing as they sometimes attend our meetings and because… as women, we are developing our area and village.” Neighbor Mother, Lupane  “Through the drama and singing that we do, we were invited to perform when we had the village and ward celebrations. This on its own shows the importance and the benefit of the group in making us known.” Neighbor Mother, Tsholotsho  “The group is recognized by the local leadership and community members. They are happy with the development because after they get knowledge there is positive change in the area. Other non-group members who are community members visit us.” Neighbor Mother, Lupane |  |
| Family Support | “Other [members] fail to come for the meetings. They face challenges from their husbands because they would be expecting them to attend some home duties.” Neighbor Mother, Lupane  “Some husbands deny us to attend or join these care groups.” Neighbor Mother, Binga  “Young mothers have a challenge from their husbands who do not agree to let them attend sessions as they will be saying it’s a waste of time since there are no benefits materially. Also, these mothers come late as they will be demotivated and mocked by their husbands at home.” Lead Mother, Lupane  “Some of their family members, even their husbands, do not want them to join care groups as they do not understand the purpose of the care group…*.*[Even] in-laws do not understand what we do in the care groups. They do not allow their daughters-in-law to join in the meetings because they want their daughters-in-law to do some household chores.” Lead Mother, Tsholotsho  “Mothers who live with their in-laws cannot enforce new laws in the household. When she is from giving birth the mother-in-law becomes in charge of the baby.” CGP, Lupane |  |

## 
